# LVTree Viewer: An Interactive Display for the All-Species Living Tree Incorporating Automatic Comparison with Prokaryotic Systematics

**DOI:** 10.1016/j.gpb.2015.12.002

**Published:** 2016-03-25

**Authors:** Guanghong Zuo, Xiaoyang Zhi, Zhao Xu, Bailin Hao

**Affiliations:** 1 T-Life Research Center, Department of Physics, Fudan University, Shanghai 200433, China; 2 Yunnan Institute of Microbiology, Yunnan University, Kunming 650091, China; 3 Thermo Fisher Scientific, South San Francisco, CA 94080, USA

**Keywords:** *Archaea* and *Bacteria*, 16S rRNA phylogeny, Monophyly, Collapsing and expanding a tree, Lineage modifications

## Abstract

We describe an interactive viewer for the All-Species Living Tree (LVTree). The viewer incorporates treeing and lineage information from the ARB-SILVA website. It allows collapsing the tree branches at different taxonomic ranks and expanding the collapsed branches as well, keeping the overall topology of the tree unchanged. It also enables the user to observe the consequence of trial **lineage modifications** by re-collapsing the tree. The system reports taxon statistics at all ranks automatically after each collapsing and re-collapsing. These features greatly facilitate the comparison of the 16S rRNA sequence phylogeny with prokaryotic taxonomy in a taxon by taxon manner. In view of the fact that the present prokaryotic systematics is largely based on 16S rRNA sequence analysis, the current viewer may help reveal discrepancies between phylogeny and taxonomy. As an application, we show that in the latest release of LVTree, based on 11,939 rRNA sequences, as few as 24 **lineage modifications** are enough to bring all but two phyla (*Proteobacteria* and *Firmicutes*) to monophyletic clusters.

## Introduction

The All-Species Living Tree project (www.arb-silva.de/projects/living-tree) is an initiative of the Editorial Office of *Systematic and Applied Microbiology* in collaboration with the ARB-SILVA (www.arb-silva.de) and List of Prokaryotic Names with Standing in Nomenclature (LPSN, www.bacterio.net) projects to construct a single phylogenetic tree for all available type strains of *Archaea* and *Bacteria* based on high-quality 16S rRNA sequences [1], [2], [3], [4]. The latest release LTPs123 (as of September 2015) contains 449 *Archaea* and 11,490 *Bacteria* sequences. However, it is a hard job to comprehend such a big tree by scrolling up and down the long PDF, especially when one wishes to compare the phylogeny with taxonomy at all ranks from phylum down to species by checking the monophyly of all the individual branches.

A similar situation has been encountered while working on the whole-genome-based Composition Vector Tree (CVTree) web server [5], [6], [7], [8] in order to enable the server to deal with many thousands of prokaryotic genomes in one run. To address this issue, several distinctive features have been introduced in the latest CVTree3 web server [8]. These features include (1) collapsing a monophyletic tree branch into a single leaf when the branch represents sequences from the same taxon according to a reference taxonomy; (2) expanding a collapsed leaf to show the constituent leaves, while the overall topology of the tree is preserved during collapsing and expanding; (3) reporting taxon statistics, *i.e.*, the number of sequences represented by each taxon, monophyletic or non-monophyletic, in the form of a hierarchical list; (4) enquiring a designated taxon by entering its name in a “Search Query” box (this is the quickest way to get to the point of interest; while the neighborhood of the enquiry is properly expanded, all the other branches are maximally collapsed); (5) making trial lineage modifications and displaying the result of re-collapsing with renewed taxon statistics; and (6) outputting print-quality sub-tree figures in different formats.

All these features have been transplanted to the LVTree Viewer presented in this study. There are some technical points in the implementation of the Viewer. For more details, the users are advised to consult the CVTree3 User’s Manual at http://tlife.fudan.edu.cn/cvtree3/.

## Description and usage of the LVTree Viewer

The LVTree Viewer is freely accessible at http://tlife.fudan.edu.cn/lvtree/ without login requirement. It takes the tree structure from LTPs123_SSU.tree.newick and lineage information from LPTs123_SSU.csv, both downloadable from the All-Species Living Tree project website. In fact, two previous releases (LTPs121 and LTPs119) are also accessible on a rotating basis.

By clicking on “Show LVTree”, a maximally-collapsed tree with two leaves, <D>Archaea{422+7} and <D>Bacteria{11156+334}, appears in the next screen. The taxon{n+m} notation is explained appropriately in what follows. Lineage information is available for each 16S rRNA sequence in LVTree. When hovering the mouse over a taxon name, a small popup window with lineage information appears for a few seconds. For example, an entry may read as “<D>Bacteria<P>Actinobacteria<C>Actinobacteria<O>Streptomycetales<F>Streptomycetaceae<G>Streptomyces<S>Streptomyces_longisporoflavus<T>Streptomyces_longisporoflavus__DQ442520…”, where <D>, <P>, <C>, <O>, <F>, <G>, <S>, and <T> stand for Domain, Phylum, Class, Order, Family, Genus, Species, and sTrain, respectively. The line above summarizes the correct lineage of a well-studied streptomycete species *Streptomyces longisporoflavus*, which was initially described by Waksman and Henrici in 1953. It will also be shown as an example of lineage modification in the later part of this study.

A standard specifier “Unclassified” is used to indicate any rank in classification lacking taxonomic assignment, *e.g.*, <O>Unclassified<F>Unclassified<G>*Vampirovibrio* gives a genus name without order and family assignment. Lineage information containing at least one specifier “Unclassified” is considered incomplete. If there are “n” entries with complete lineage information and “m” entries with incomplete or missing information for a given taxon, these numbers appear as taxon{n+m}. Furthermore, the two lines <D>Archaea{422+7} and <D>Bacteria{11156+334} are shown in red, because both are monophyletic, whereas non-monophyletic entries would appear in blue. An entry can be expanded by clicking on the solid circle in front, whereas an entry with no circle present contains only one single sequence and thus cannot be further expanded. In a screen with a partially-expanded tree one may click on a blank circle to have the subordinate branches collapsed.

**Figure 1:**
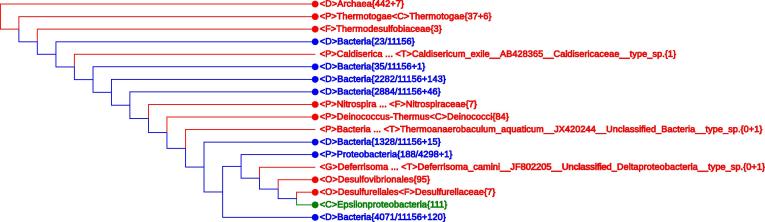
**A collapsed All-Species Living Tree with the neighborhood of *Epsilonproteobacteria* expanded** The tree shown here is the whole LVTree representing all 11,939 16S rRNA sequences. Monophyletic and non-monophyletic taxa are indicated in red and blue, respectively. Taxon retrieved when performing “Search Query” with “Epsilonproteobacteria” is indicated in green. “Unclassified” indicates missing classifier. Lineage information containing one or more “Unclassified” is considered incomplete. The {n+m} notation indicates that there are n genomes with complete lineage information and m genomes with incomplete or missing lineage information. {n+m} is indicated as {n} when m = 0, while when n = 0, {n+m} is indicated as {0+m}. <D>, <P>, <C>, <O>, <F>, <G>, <S>, and <T> stand for domain, phylum, class, order, family, genus, species, and strain, respectively.

In the preamble line of a display screen, four boxes are labeled as “Top Node”, “Lineage Modification”, “Monophyly List”, and “Search Query”. By clicking on “Monophyly List”, a hierarchical summary of all taxa with statistics appears with “Yes” denoting a monophyletic taxon and “No” denoting a non-monophyletic taxon, respectively. There are four options to present the summary: “Total” shows statistics of all taxa; “Monophyly” gives a list of all monophyletic taxa (since any taxon represented by a single sequence is trivially “monophyletic”, one can hide such entries to get a more comprehensible list); “None” lists the non-monophyletic taxa; and “Unclassified” shows entries with at least an “Unclassified” specifier in the lineage information, *i.e.*, those entries that correspond to the augend “m” in the taxon{n+m} notation. The quickest way of getting to a taxon of interest is using the “Search Query” function. For example, typing “Epsilonproteobacteria” to replace “Search Query” in the box would lead to a collapsed tree with <C>Epsilonproteobacteria{111} displayed in green, indicating that this class represented by 111 rRNA sequences has already been well defined, as shown in Figure 1. The “Top Node” pull down list is used to restore the displayed tree to a designated node as the leftmost node after collapsing.

The present prokaryotic taxonomy as collected in Bergey’s Manual [9] and LPSN [10] is largely based on 16S rRNA sequence analysis. Ideally speaking, one should expect full agreement of the branching order in LVTree with the taxonomic hierarchy. Given an input dataset and a well-tested method of phylogeny inference, the LVTree must be considered as a fixed subject that cannot be adjusted or modified. To the contrary, prokaryotic taxonomy has always been a work in progress. Therefore, discrepancies revealed when comparing LVTree with systematics most probably hint on problems in taxonomic assignments. However, there might be errors such as mislabeling of sequences in the underlying LVTree dataset. The LVTree Viewer may help spot some of the errors as well.

The LVTree Viewer provides a mechanism to examine the consequence of trial lineage modifications. A user may submit a Lineage Modification file, in which each entry describes a proposed modification: old_lineage<space>new_lineage.

When suggesting a lineage modification, the names under <S> and <T> are always kept unchanged. In doing so, no confusion would occur concerning the published or cited species names, which can always be used to do “Query Search”. In other words, species and strain names in a collapsed tree appear in the same way as in the original LVTree.

By clicking on “Lineage Modification” an empty window appears. A user may type in own modifications or drag in a prepared file from one’s local computer. Alternatively, an example Lineage Modification file (File S1) may be invoked. Upon clicking on “Submit”, it takes a while for the system to display the re-collapsed tree and the re-calculated Monophyly List. This process may be carried out repeatedly and one can “Save” the Lineage Modification file to the local computer for future use. By clicking on “Clear Text” and then “Submit”, one may undo the modifications. The Project number given at the beginning of the job session becomes useful, when the user has formed a Lineage Modification file. By typing in the Project number when starting a new LVTree session, the Lineage Modification file is called back from the user’s Workspace, which is kept for 7 days after the last run.

When accessed for the first time, the LVTree Viewer comes with a default Lineage Modification file (File S1). In order to check the modifications suggested in the subsequent Sections, the user should disable this file by using the “Clear Text” and “Submit” buttons. In other words, the following discussions aim at a “bare” viewer without making any lineage modification.

## Lineage modifications that bring all but two phyla monophyletic

The notion of monophyly serves as a basic guiding principle when comparing phylogenetic tree with reference taxonomy. As an application of the LVTree Viewer, we check the monopoly of all branches at the rank phylum.

An inspection of the Monophyly List shows that 21 phyla appear as monophyletic clusters without making any lineage modifications. These include Aquificae{27+1}, Armatimonadates{3}, Caldiserica{1}, Chlamydiae{13}, Chloroflexi{33}, Chrysiogenetes{4}, Crenarchaeota{51}, Cyanobacteria{15}, *Deinococcus-Thermus*{84}, Dictyoglomi{2}, Euryarchaeota{371+6}, Fibrobacteres{4}, Gemmatimonadates{1}, Lentisphaera{4}, Nitrospira{7}, Planctomycetes{23}, Spirochaetes{92+1}, Thaumarchaeota{1}, Thermodesulfobacteria{8}, Thermotogae{43}, and Verrucomicrobia{41+2}.

By typing the non-monophyletic phylum names into the “Search Query” box one by one, it is easy to spot a small number of outliers that violate monophyly of the corresponding phyla. Examining the neighborhood of a given outlier, in combination with tracing back the history of taxonomic assignment, helps to spot a sequence error or suggest a proper lineage modification.

**Figure 2:**
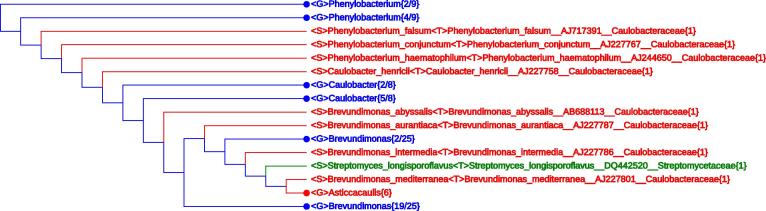
**A sequence with erroneous lineage information appeared under the name of *Streptomyces longisporoflavus*** The sequence carrying the name of the actinobacterium *Streptomyces longisporoflavus* gets deeply into the family *Caulobacteraceae* in the order *Rhizobiales* of phylum *Proteobacteria*, indicating that there must be an error with the sequence used (see the “Lineage modifications that bring all but two phyla monophyletic” section for more details). Taxon retrieved when performing “Search Query” with “<P>*Actinobacteria*” is indicated in green. Please see the legend of Figure 1 for the definition of other indications.

To demonstrate this function, “<P>Actinobacteria” is typed into the “Search Query” box. A big cluster in green with a single branch in red appears when scrolling down the tree display. This represents the main body of the phylum *Actinobacteria* with an “invading” species “<S>Flavobacterium oceanosedimentum” from the phylum *Bacteroidetes* (data not shown). At the same time five species escape from *Actinobacteria* into other phyla, including <S>*Streptomyces longisporoflavus* as shown in Figure 2.

Apparently, a problem is encountered. The genus *Streptomyces* and the species *S. longisporoflavus* were proposed in 1943 and 1953 by Waksman and coworkers. Nowadays *Streptomyces* is a large genus containing 669 species and 38 subspecies as listed in LPSN [10]. Being a long-studied species of medical significance, the taxonomic assignment of *S*. *longisporoflavus* is beyond all doubt. Morphologically *S*. *longisporoflavus* is clearly distinct from *Brevundimonas* species, so the confusion can only stem from the 16S rRNA sequence (GenBank accession No. DQ442520) deposited by Goodfellow and colleagues in 2006. Fortunately, they deposited another 16S rRNA sequence (GenBank accession No. NR_115963) for this species in 2015. A BLAST comparison shows that NR_115963 has high similarity with 16S rRNA sequences from other *Streptomyces* species, while DQ442520 is close to 16S rRNA sequences from *Brevundimonas*. Most probably, DQ442520 represents a mislabeled *Brevundimonas*. Since LVTree users cannot change or replace the sequence database, we propose the following lineage modification just to “hide” the inconsistency:

<P>Actinobacteria<C>Actinobacteria<O>Streptomycetales<F>Streptomycetaceae<G>Streptomyces<S>Streptomyces_longisporoflavus
<P>Proteobacteria<C>Alphaproteobacteria<O>Rhizobiales<F>Claulobacteraceae<G>Brevundimonas<S>Streptomyces_longisporoflavus (please note that in between the two seemingly lines above, there is only a <space> character, not a carriage return).

Similarly, *Thermoleophilum minutum*, another outlier from *Actinobacteria*, also has two different 16S rRNA sequences present (GenBank accession Nos. HQ223108 in LVTree and AJ458464 in Vol. 5, the Bergey’s Manual [9], respectively), leading to inconsistent taxonomic placement. This is treated analogously by a temporal lineage modification (File S1). The genus *Thermoleophilum* was proposed in 1986 with two species described so far (*Thermoleophilum album* and *T. minutum*). New family, order, and class were introduced for this genus in 2005, 2009, and 2013, respectively. In LVTree, while *T. album* remains in the phylum *Actinobacteria*, *T. minutum* gets deeply into <G>*Pseudomonas* in <P>*Proteobacteria*.

**Figure 3:**
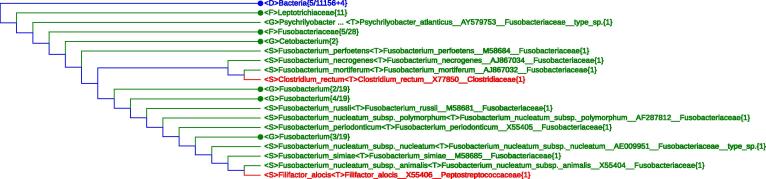
***Clostridium rectum* and *Filifactor alocis* violate monophyly of the phylum *Fusobacteria*** The two monophyletic branches (red) appear when performing “Search Query” with “Fusobacterium”, demonstrating how the color usage helps to single out “outliers” or “invaders” in tree branches. Taxa retrieved when performing “Search Query” with “Fusobacterium” are indicated in green. Please see the legend of Figure 1 for the definition of other indications.

Another apparent sequence error happens to *Filifactor alocis*, a *Firmicutes* species getting into the very depth of the phylum *Fusobacteria* (Figure 3). The 16S rRNA sequence (GenBank accession No. X55406) chosen by LVTree was published among a group of 14 members of the genus *Fusobacterium* in 1991 by Lawson et al [11]. However, it was soon pointed out that “Lawson et al have been erroneously sequenced some other *Fusobacterium* species instead of *Fusobacterium alocis* 35896T” [12] and the species was reclassified as *Filifactor alocis* to the phylum *Firmicutes*. In other words, X55406 is associated with some other strain of *Fusobacterium*, but Lawson et al simply changed its name to *Filifactor alocis* when submitting the 16S rRNA sequence in 2004, causing the inconsistency revealed here. Therefore, the LVTree dataset should exclude X55406 from the *Fusobacterium* group and use the sequence with GenBank accession No. AJ006962 (indicated in Vol. 3, the Bergey’s Manual [9]) for *Filifactor alocis*. By the way, AJ006962 still carries a wrong sequence name *Fusobacterium alocis* but with a correct organism source *Filifactor alocis.* The suggested lineage modification is a compromise before appropriate correction is made in LVTree.

We skip further discussions and list briefly all suggested lineage modifications which are enough to bring all but two phyla (*Proteobacteria* and *Firmicutes*) to monophyletic clusters.

**Figure 4:**
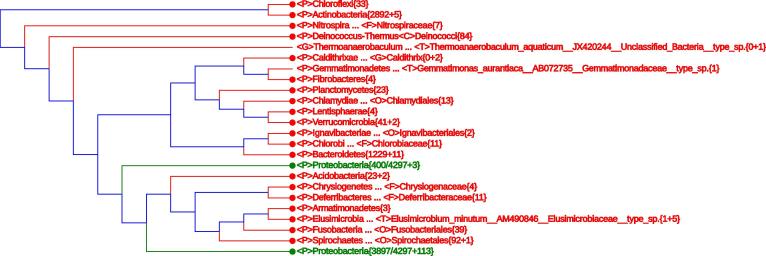
**A single LVTree branch accommodating 8959 16S rRNA sequences** The branch comprises 21 well-defined phyla, an “Unclassified_Bacterium” at phylum level, and the phylum *Proteobacteria* split into two clusters. Taxa retrieved when performing “Search Query” with “*Proteobacteria*” are indicated in green. Please see the legend of Figure 1 for the definition of other indications.

1. *Arthrobacter* is a genus proposed in 1947 and the species *Arthrobacter viscosus* was first described in 1965. It inherits the phylum assignment to *Actinobacteria* from the genus name, but in LVTree it gets into the depth of the genus *Rhizobium* within the class *Alphaproteobacteria*. Its taxonomic position was questioned by Keddie et al in 1986 (Vol. 2, the 1st Edition of Bergey’s Manual). Its 16S rRNA sequence that was published in 2005 [13] showed a high degree of similarity to several *Rhizobium* species (97.6%–98.7%). The suggested lineage modification takes these facts into account.
2. *Brevibacterium* is a genus proposed in 1953 and the two species *Brevibacterium halotolerans* and *Brevibacterium frigoritolerans* were described in 1967, inheriting the phylum assignment from the genus name. In LVTree, these two species locate unquestionably in the genus *Bacillus*, far apart from the main cluster made of 30 *Brevibacterium* species. In fact, when initially submitted in 2007, 16S rRNA sequences (accession Nos. AM747812 and AM747813) were assigned a *Bacillus* lineage in GenBank. Therefore, the suggested lineage modifications just recollect this overlooked fact in the LVTree database. As a result, these modifications lead to a monophyletic <G>*Brevibacterium*{30} in LVTree.
3. The genus *Acetobacterium* and species *Acetobacterium flavidum* were proposed in 1984 [14]. A second species *Acetobacterium faecale* was proposed in 1987 [15]. However, it was stated on page 43 of Vol. 4, the Bergey’s Manual, that “placement of *Acetomicrobium* as a member of the family *Bacteroidaceae* is not certain because of the lack of rRNA gene sequence data.” In fact, the type strain of *A. flavidum* bears 99.7% ([4]) or 99.8% ([16]) 16S rRNA sequence similarity with *Anaerobaculum mobile*, the sixth sequenced species in a newly-proposed phylum *Synergistetes* [17]. In addition, the 16S rRNA sequence of *A. faecale* “shows an unexpected affiliation with a deep branch in *Clostridia* (99.46% similarity against *Caldicoprobacter oshimai*)” [18]. The suggested lineage modifications take these facts into account and effectively render *Acetomicrobium* an empty genus.
4. *Anaerorhabdus furcosa* was reclassified from the genus *Bacteroides* in 1986 [19] as the only member of a new genus, but the phylum assignment of the latter has not been questioned then. Therefore, it is still classified under *Bacteroidetes* in the original LVTree lineage. However, previous study indicates an unexpected affiliation of this species with the family *Erysipelotrichaceae* (91.6% similarity with *Holdemania filiformis*) [4]. In fact, in LTPs123, two other neighbors of *A. furcosa*, *Solobacterium* and *Bulleidia*, appear even closer to *Erysipelotrichaceae*. The proposed lineage modification reflects this fact.
5. *Bacteroides cellulosolvens* has been reclassified to a new genus in the class *Clostridia* as *Pseudobacteroides cellulosolvens* in 2014 [20]. However, both *B. cellulosolvens* and *P. cellulosolvens* appear juxtaposed to each other in LVTree. It seems that it is appropriate to delete the one misclassified to *Bacteroidaceae*.
6. *Bacteroides coagulans*, a species described in 1933, locates deeply within class *Clostridia* of phylum *Firmicutes*, far from the phylum *Bacteroidetes* in LVTree. In EzTaxon-e [21] it is assigned to a newly-proposed genus *Ezakiella* [22] under *Incertae sedis* family XI. Our suggested lineage modification follows EzTzxon-e with <F>Unclassified, though the re-collapsed LVTree hints on its belonging to the family *Peptoniphilaceae*.
7. The description of both *Bacteroides galacturonicus* and *Bacteroides pectinophilus* in 1986 was based on phenotypic data [23]. The 16S rRNA sequences were deposited to GenBank (accession Nos. DQ497993 and DQ497994) in 2006 with mentioning of “reclassification” in the unpublished title, but only one lineage has been changed to *Clostridiales*. Their positions in LVTree show that these strains should be assigned to the family *Lachnospiraceae* in two different genera. This fact is reflected in the suggested lineage modifications. It is of note that the whole-genome sequence (GenBank accession No. ABVQ01000036) instead of the 16S rRNA sequence (DQ497993) was given in the strain tag of *B. pectinophilus* in LVTree*.*
8. Type strain of *Flavobacterium oceanosedimentum* shows an unexpected 99.8% similarity to *Curtobacterium citreum* [4]. Although the association of this species with the genus *Flavobacterium* was questioned in the 1st edition of the Bergey’s Manual in 1984, formal proposal to transfer it to the genus *Curtobacterium* in the phylum *Actinobacteria* appeared only in 2009 [24]. Such an assignment was adopted in Vol. 5, the Bergey’s Manual [9], which is reflected in the proposed lineage modification. The modification leads to a monophyletic branch *Curtobacterium*{8} in LVTree, and the monophyly would be violated if *F. oceanosedimentum* was miss-classified to *Flavobacterium*.
9. The 16S rRNA sequences of the two *Caldithrix* species were deposited in 2003 [25] (*Caldithrix abyssi*) and 2010 [26] (*Caldithrix palaeochoryensis*), respectively, without proper lineage information except for the genus name. Their strain tags in LVTree carry a specifier “Unclassified-Deferribacterales”, but actually they take the position of a separate phylum in LVTree (Figure 4). On the Integrated Microbial Genomes (IMG) website (img.jgi.doe.gov), they are given a new phylum name *Caldithrixae*, not listed anywhere else in the literature. The suggested lineage modification adopts this phylum assignment with “Unclassified” lower ranks.
10. In LVTree, the 16S rRNA sequence (GenBank accession No. RF733681) of *Ilyobacter delafieldii* escapes from the phylum *Fusobacteria* and appears as a close sister to *Clostridium homopropionicum* with 98.8% sequence similarity as described previously [4]. The latter is attributed to the genus *Clostridium senso stricto*. Therefore, the suggested modification puts the species *I. delafieldii* in the *Clostridium* lineage.
11. The species *Fusobacterium naviforme* was described in 1909 and the genus was introduced in 1922. In LVTree *F. naviforme* appears to be the closest neighbor of *Moryella*, a single-species genus in *Lachnospiraceae* in the phylum *Firmicutes*. The lineage modification follows EzTaxon-e [21] and assigns *F. naviforme* to the genus *Moryella*.
12. *Clostridium rectum* was attributed in 1922 to the old genus *Clostridium* Prazmowski 1880, long before the rRNA concept appeared. In 1994 it was recognized that “although *C. rectum* forms endospores, it is clearly a member of the genus *Fusobacterium*” [27]. The 16S rRNA sequence was deposited in 2004 with a conditional organism name [*Clostridium*] *rectum*. The suggested lineage modification simply recognizes this consensus. After making the modifications explained in this and the previous entry, there appears a monophyletic genus *Fusobacterium*{20} in the re-collapsed LVTree.
13. The genus *Acetobacter* was described in 1898 with type species *Acetobacter acetis* discovered by Pasteur in 1864*.* The bacterium *Acetobacter pasteurianus* subsp. *ascendens* has been well-studied since 1898*.* In LVTree it locates within the genus *Lysinibacillus* in the phylum *Firmicutes*, while the other two subspecies strains of *Acetobacter pasteurianus* remain in the designated phylum *Proteobacteria*. The suggested lineage modification assigns it to *Firmicutes*.
14. *Gemmiger formicilis* is a species described in 1975 [28]*.* The 16S rRNA sequence of the type strain bears 97.5% similarity with that of *Subdoligranulum variabile* in the family *Ruminococcaceae* [4]. Both *Gemmiger* and *Subdoligranulum* are single-species genera for the time being. The suggested lineage modification temporarily puts *G. formicilis* in the genus *Subdoligranulum.*
15. The bacterium *Vampirovibrio chlorellavorus* was first described as a member of the genus *Bdellovibrio* in 1972 [29] and a new genus was proposed for it in 1980 [30]. In LVTree, it is next to the phylum *Cyanobacteria*{10}, as if it was a cyanobacterium or represents an yet uncharacterized phylum. Although the suggested lineage modification attributes it to an “Unclassified” phylum, LVTree Viewer’s re-collapsing mechanism automatically absorbs it into *Cyanobacteria*. In view of the insufficient representation (only 15 among the 11,939 sequences) and the even less-studied taxonomy of *Cyanobacteria*, this problem awaits further scrutiny. In addition, we note that the order *Bdellovibrionales* turns out to be an outlier from the phylum *Proteobacteria* in whole-genome-based CVTree [8].
16. In LVTree, both the single-species genera *Asteroleplasma anaerobium* [31] and *Haloplasma contractile* [32] fall in a “grey area” between the *Firmicutes* and *Mollicutes*. *Mollicutes* was taken out of *Firmicutes* as a separate phylum in Vol. 4, the Bergey’s Manual [9]. This area predominantly consists of species from *Erysipelothrichaceae*, the only family in the class *Erysipelotrichia*. As for *A. anaerobium*, “the question of possible monophyly and the phylogenetic position of *Asteroleplasma* with respect to other *Mollicutes* remains open” (Vol. 4, the Bergey’s Manual, page 723). The branching position of *H. contractile* “was equidistant to the taxa considered to be representative lineages of the phyla *Firmicutes* and *Tenericutes*” [32].
17. In the latest version LTPs123 of September 2015, there appeared a “new” species named *Chloracidobacterium thermophilum* whose whole genome was released in 2007 under the name *Candidatus* Chloracidobacterium thermophilum B [33]. The *Candidatus* status has prevented it from being included into the previous releases of LVTree until EMBL made a change recently. However, in LVTree *C. thermophilum* gets immersed in the phylum *Elusimicrobia* in contradiction to the original taxonomic assignment to phylum *Acidobacteria*. As CVTree [8] also clearly supports its belonging to *Acidobacteria*, it is suspected that a wrong sequence has been used in LVTree. Indeed, the GenBank accession number CP002514 cited in LVTree corresponds to a whole genome instead of a 16S rRNA. Hopefully, this point will be clarified in the next release of LVTree.

Given 11,939 of 16S rRNA sequences in total, it is remarkable that so few lineage modifications, caused either by sequence error or by taxonomic misplacement, are capable to bring all but two prokaryotic phyla to monophyletic clusters. Furthermore, it is not surprising that most of the suggested modifications are introduced for taxa proposed in the pre-rRNA era. Some modifications simply hint on necessary taxonomic revisions according to modern 16S rRNA sequence analysis. However, we refrain from making formal emendation for the time being in order to wait for more phenotypic and phylogenetic support.

## Phylogeny and taxonomy at the phylum level

**Figure 5:**
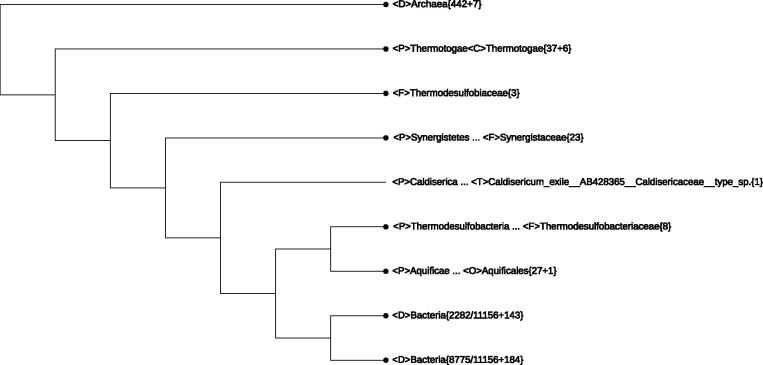
**An appropriately-collapsed LVTree based on all 11,939 16S rRNA sequences** This tree was obtained by making only one lineage modification bringing *Acetomicrobium flavidum* from the phylum *Bacteroidetes* to the phylum *Synergistetes* (see Point 3 in Section “Lineage modifications that bring all but two phyla monophyletic” for more details). The fractions 2282/11156 and 8775/11156 in the last two branches mean 2282 and 8775 out of totally 11,156 genomes with lineage information shown in the whole tree, respectively. Please see the legend of Figure 1 for the definition of other indications.

The aforementioned lineage modifications have added 11 monophyletic phyla. These include *Acidobacteria*{23+2}, *Actinobacteria*{2892+5}, *Bacteroidetes*{1229+11}, *Caldithrixae*{2}, *Chlorobi*{11}, *Deferribacteres*{11}, Elusimicrobia{1+5}, *Fusobacteria*{39}, *Ignavibacteriae*{2}, *Synergistetes*{23}, and *Tenericutes*{186}. Only two phyla, *Firmicutes*{2090+134} and *Proteobacteria*{4297+116}, remain non-monophyletic. Being “big” divisions, the last two phyla accommodate 56% of the 16S rRNA sequences in the LVTree dataset. In addition, a single species without proper phylum assignment (*Thermoanaerobaculum aquaticum*) takes the position of a separate phylum. Figure 5 shows the whole LVTree in an appropriately-collapsed form. This tree may be further scrutinized in three steps.

The first step deals with the upper 7 branches, which include 8 well-defined phyla (3 archaeal phyla collapsed into one leaf and 5 bacterial phyla) and a single family <F>*Thermodesulfobiaceae*{3} taking the status of phylum. Only three species are listed in this family in LPSN [10] and Vol. 3, the Bergey’s Manual [9]. These include *Thermodesulfobium narugense*, *Coprothermobacter proteolyticus*, and *Coprothermobacter platensis*. A 16S rRNA survey was performed in 2004 [34] and recognized *Coprothermobacter* as an “established phylum”. Furthermore, “*T. narugense* is sufficiently isolated from previously described bacteria to justify creation of a new phylum” (Vol. 3, the Bergey’s Manual, page 1269). The whole-genome-based CVTree analysis also supports *Coprothermobacter* to acquire the status of a separate phylum [35].

<D>Bacteria(2282/11156+143) makes the second step of investigation which invokes the most taxonomically-confused branch. The third step starts by expanding the last branch <D>Bacteria{8775/11156+184} to a monophyletic branch made of 21 well-defined phyla, in addition to a split phylum *Proteobacteria*, and a single species *T. aquaticum* (Figure 4). The last species takes the status of a new phylum, though the original report [36] puts it in *Acidobacteria* Subdivision 23. Although the same 16S rRNA sequence (GenBank accession No. JX420244) is used in [36] and in LVTree, the conclusion is clearly different. Since the whole-genome-based CVTree approach [8] puts *Thermoanaerobaculum aquaticus* in the phylum *Acidobacteria* [37] in agreement with [36], the LVTree placement of this species requires further examination.

Traditionally, the phylum *Proteobacteria* contains five classes, from *Alphaproteobacteria* to *Epsilonproteobacteria*. In recent years, a few new classes have been proposed, for example, *Zetaproteobacteria* [38], *Acidithiobacillia* [39], and *Oligoflexia* [40], [41]. In LVTree, instead of forming a monophyletic branch, *Proteobacteria* splits into two disjointed clusters.

The first cluster contains the *Alpha-*, *Beta-*, *Gamma-*, and *Zeta-proteobacteria*, as well as the class *Acidithiobacillia*, representing altogether 4010 rRNA sequences. A few lineage modifications would make both *Alpha-* and *Beta-* groups monophyletic. Carl Woese and coworkers observed in the mid 1990s [42] that the *Beta*- group gets inserted into *Gamma*- and these two groups, taken together, make a larger monophyletic cluster. This situation holds true if the newly-proposed class *Acidothiobacillia* [39] is considered a part of the *Gamma*-group as it was before. The second cluster comprises the *Delta-* and *Epsilon-proteobacteria*, as well as the newly-proposed *Oligoflexia* classes, representing 396 rRNA sequences in total. While <C>Epsilonproteobacteria{111} comes out as a monophyletic group from the outset, all species in the *Delta*-group appear to be intermixed. Besides the questionable class status of *Oligoflexia*, the whole cluster may be called a *Delta*/*Epsilon-*group.

Now we arrive at the most intricate part of Figure 5, namely, what was obtained by expanding the branch <D>Bacteria(2282/11156+143). Except the three monophyletic phyla *Dictyoglomi*{2}, *Cyanobacteria*{6+9}, and *Tenericutes*{186}, all other sequences come from the phylum *Firmicutes* with many taxonomic uncertainties. Actually, the branching order in this part challenges the phylum status of all the four named divisions, including *Cyanobacteria* whose taxonomy has been a long-due problem, partially because it has been treated under the Botanic Code and partially owning to the under-representation of 16S rRNA sequences in LVTree.

Historically, many phyla have been extracted from the phylum *Firmicutes*. For instance, at least four other phyla were recognized within *Firmicutes* [43]. *Actinobacteria* was an order within *Firmicutes* in the first edition of Bergey’s Manual, and then promoted to a class in mid 1990s, and, finally, to a separate phylum in 2012 (Vol. 5, the Bergey’s Manual), whereas *Tenericutes* changed its class status in *Firmicutes* to an individual phylum in 2010 (Vol. 4, the Bergey’s Manual). Yet *Firmicutes* remains a warehouse for new higher-rank taxa.

In a sense, taxonomic problems of prokaryotes are now concentrated in just one line of Figure 5 as discussed in the two paragraphs above. Since the aim of this paper is to introduce a convenient tool for studying LVTree, the discussion of these classification problems would take us too far afield. However, the combined use of 16S rRNA sequence analysis by the present LVTree Viewer and the whole-genome-based CVTree web server [6], [7], [8] will surely bring prokaryotic phylogeny and taxonomy to a much better shape. However, we emphasize that all the lineage modifications suggested in this paper only serve as demonstration of how to use the interactive tree display. Any actual taxonomic revision must follow the International Code for Bacterial Nomenclature [44], [45] and be published in an appropriate journal.

## Authors’ contributions

BH conceived the idea of the work. GZ designed and implemented the LVTree Viewer. GZ, XZ, and ZX collected sequence data of species under question. BH and XZ carried out the taxonomic comparison and suggested most of the lineage modifications. All authors read and approved the final manuscript.

## Competing interests

The authors have declared that no competing interests exist.

## Supplementary material

Supplementary File S1 Examples of lineage modifications. A list of the suggested lineage modifications that would bring all but two phyla into monophyletic clusters is provided. A few additional modifications have been included with brief notes or references in the form of comments with a leading pound sign #. These modifications concern, in particular, the subclass and suborder ranks in the phylum *Actinobacteria* that have been promoted to classes and orders in Vol. 5, the Bergey’s Manual [9]. Some modifications deal with obvious omissions or inexactitudes in the original lineage information. Most importantly, this file contains 24 modifications that bring all but two phyla to monophyletic clusters. Please note all these suggestions only serve as demonstration of how to use the interactive tree display. Any actual taxonomic revision must follow the International Code for Bacterial Nomenclature [44], [45] and be published in an appropriate journal.
